# Chloroplast genomes in seven *Lagerstroemia* species provide new insights into molecular evolution of photosynthesis genes

**DOI:** 10.3389/fgene.2024.1378403

**Published:** 2024-04-02

**Authors:** Ling He, Sujuan Xu, Xinnian Cheng, Hanlin Huang, Hongyu Dai, Xin Wang, Zhiyang Ding, Ming Xu, Haoran Gu, Na Yan, Chunyan Wang

**Affiliations:** ^1^ College of Horticulture and Landscape Architecture, Jinling Institute of Technology, Nanjing, China; ^2^ College of Horticulture, Nanjing Agricultural University, Nanjing, China; ^3^ College of Medicine, Southeast University, Nanjing, China

**Keywords:** *Lagerstroemia indica*, chloroplast genome, photosynthesis genes, molecular evolution, climatic adaptation

## Abstract

*Lagerstroemia indica* is an important commercial tree known for the ornamental value. In this study, the complete chloroplast genome sequence of *Lagerstroemia indica* “Pink Velour” (*Lagerstroemia* “Pink Velour”) was 152,174 bp in length with a GC content of 39.50%. It contained 85 protein coding genes (PCGs), 37 tRNAs, and 8 rRNA genes. 207 simple sequence repeats (SSRs) and 31 codons with relative synonymous codon (RSCU)value > 1 were detected. Phylogenetic analysis divided 10 *Lagerstroemia* species into evolutionary branches of clade A and clade B. We conducted a comparative analysis of *Lagerstroemia* “Pink Velours” complete chloroplast genome with the genomes of six closely related *Lagerstroemia* species from different origins. The structural features of all seven species were similar, except for the deletion of ycf1 nucleobases at the JSA boundary. The large single-copy (LSC) and the small single-copy (SSC) had a higher sequence divergence than the IR region, and 8 genes that were highly divergent (*trnK-UUU*, *petN*, *psbF*, *psbJ*, *ndhE*, *ndhD*, *ndhI*, *ycf1*) had been identified and could be used as molecular markers in future studies. High nucleotide diversity was present in genes belonging to the photosynthesis category. Mutation of single nucleic acid was mainly influenced by codon usage. The value percentage of nonsynonymous substitutions (Ka) and synonymous substitutions (Ks) in 6 *Lagerstroemia* species revealed that more photosynthesis genes have Ka or Ks only in *Lagerstroemia fauriei*, *Lagerstroemia limii*, and *Lagerstroemia subcostata*. These advances will facilitate the breeding of closely related *Lagerstroemia* species and deepen understanding on climatic adaptation of *Lagerstroemia* plants.

## 1 Introduction

The ornamental plant *Lagerstroemia indica* (crape myrtle), belonging to *Lagerstroemia* of Lythraceae, is a perennial deciduous tree (Li and Liang, 2021). *Lagerstroemia indica,* native to the southeastern part of Asia, is a famous flowering tree that is widely cultivated in landscapes (Wang et al., 2011). Apart from its ornamental value, *L. indica* also possesses air-purifying properties, effectively absorbing smoke and dust particles (Guo et al., 2023). Additionally, certain species of *L. indica hold* commercial significance due to their wood and medicinal properties (Xu et al., 2017; Zheng et al., 2020). Previous research on *L. indica* mainly covered propagation and breeding (Wang et al., 2021; Huang et al., 2022; Jiang et al., 2023). The propagation of *L. indica* typically involves the use of true-seedling derived from seed, cutting, and tissue culture (Wang et al., 2021; Huang et al., 2022; Jiang et al., 2023). However, the differentiation of offspring traits among true-seedlings can often be significant. Cutting and tissue culture are rapid and economical propagation methods that stably maintain the characteristics of the plant (Pérez-Molphe-Balch et al., 2015). Research on cutting and tissue culture primarily focuses on the type and concentration of hormones, growth media, and the degree of lignification of branches (Huang et al., 2022). In the process of breeding, the source of parents and the distance of genetic relationships cannot be distinguished, which necessitates much follow-up work because of variability in bred offspring. Deeper molecular identification can solve the above problems (Feng et al., 2018). Although previous studies have reported the assembly of the chloroplast genome, chloroplast genome characteristics, and phylogenetic analysis of *L. indica*, the specific evolutionary mechanisms of this species remain largely unexplored (Xu, et al., 2017; Zheng, et al., 2020; Wang, et al., 2023).

A set of genes related to photosynthesis, energy metabolism, protein synthesis, and nitrogen and sulphur assimilation were identified in plant chloroplasts, with characteristics of conservative and maternal inheritance (Pogson et al., 2015). Chloroplast genomes in angiosperms exhibit a conserved circular structure comprising four regions: a large single-copy (LSC) region, two inverted repeats (IRA/IRB), and a small single-copy (SSC) region (Asaf et al., 2017). Chloroplast genomes have played a crucial role in elucidating evolutionary relationships within phylogenetic clades and uncovering substantial variation in sequence and structure among plant species (Daniell et al., 2016). In recent years, the rapid advancements in chloroplast genome sequencing research have opened up new avenues for investigating molecular identification, genetic relationships, and phylogenetic evolution in ornamental plants. Significant progress has been made in obtaining complete chloroplast genome sequences for various woody flowering plants, including Rosa (Gao et al., 2023), Prunus (Abdulrahman et al., 2023), and Paeonia (Wu et al., 2020). In this study, we present the complete chloroplast genome of the cultivar *Lagerstroemia* “Pink Velour” and perform comparative chloroplast genome analyses using six previously published *Lagerstroemia* species obtained from the National Center for Biotechnology Information (NCBI) organelle genome database (https://www.ncbi.nlm.nih.gov). Selection may be driving the promotion of amino acid diversity in certain genes, thereby enabling rapid adaptation to environmental changes, such as varying light intensity levels (Gao et al., 2019). Photosynthetic plants commonly exhibit a pattern of slow evolutionary rates for photosynthesis genes, including psbN, psbI, psaC, atpH, petD, psbD, and psbM. In contrast, genes involved in replication (rpl2, rpl20, and rpl23), photosynthesis (rbcL and psbJ), genes with unknown functions such as ycf4, and other genes like ccsA evolved more rapidly, resulting in higher Ka/Ks values (Gu et al., 2019; Zheng et al., 2020; Wang et al., 2023). Our analyses encompass chloroplast genome organization, identification of simple sequence repeats (SSRs), codon usage, phylogenetic relationships, patterns of nucleotide substitutions, and the evolution of photosynthesis genes. This study will facilitate the breeding of closely related *Lagerstroemia* species by enabling valuable gene identification and especially by deepening our understanding of genetic and evolutionary significance, which provides information on climatic adaptation in important ornamental plants.

## 2 Materials and methods

### 2.1 Plant materials

For this study, fresh young leaves were carefully collected from three one-year-old *L. indica* “Pink Velour” plants derived from cutting seedlings at Jingling Institute of Technology (Nanjing, China). To preserve the genetic material, the collected samples underwent thorough washing and were promptly frozen in liquid nitrogen until DNA extraction. Chloroplast genomes of six distinct *Lagerstroemia* species were acquired from the NCBI organelle genome database (https://www.ncbi.nlm.nih.gov) to serve as comparative references.

### 2.2 DNA extraction and sequencing

Chloroplast genomic DNA extraction of *L. indica* “Pink Velour” was conducted using a kit designed for plant chloroplast genomic DNA isolation, which was obtained from Beijing Kangwei Century Company (CwBio, Beijing, China). Assessment of DNA quality involved agarose gel electrophoresis and a NanoDrop-2000 micro-spectrophotometer (Thermo Fisher, America). Following the qualification of the samples, a sequencing library featuring an insertion fragment size of 350 bp was constructed. Short paired-end libraries were prepared, and Nanjing Jisi Huiyuan Biotechnology Co. (Nanjing, China) executed the sequencing on the Illumina NovaSeq 6,000 platform, with a read length of 150 bp. Filtering of low-quality raw data (raw reads) was accomplished using Fastp v0.20.0 (https://github.com/OpenGene/fastp) software, which involved truncating adaptors and primer sequences in reads, and removing low-quality reads (Qphred≤5).

### 2.3 Chloroplast genome assembly and annotation

For *de novo* assembly (Bankevich et al., 2012), SPAdes v3.10.1 software (http://cab.spbu.ru/software/spades/) was employed with default parameters. Prodigal v2.6.3 (https://www.github.com/hyattpd/Prodigal) facilitated the annotation of protein-coding genes (Hyatt et al., 2010), while HMMER v3.1b2 (http://www.hmmer.org/) and Aragorn v1.2.38 (http://www.ansikte.se/ARAGORN/) were utilized for annotating RNAs and tRNAs in the chloroplast genomes of the six species (Xie et al., 2021). Secondly, based on the closely related species already published on NCBI, their gene sequences were extracted. Then, blast v2.6 (https://blast.ncbi.nlm.nih.gov/Blast.cgi) was used to align the assembled sequences, resulting in the second annotation result. Manual inspection of the genes with differences between the two annotation results was then conducted to remove incorrect and redundant annotations, and determine the boundaries of multiple exons, thereby obtaining the final annotation. Subsequently, OGDRAW (https://chlorobox.mpimp-golm.mpg.de/OGDraw.html) software was employed to generate physical maps of the chloroplast genomes (Lohse et al., 2007).

### 2.4 Codon usage

Relative synonymous codon usage (RSCU) data were acquired using the DAMBE v6.04 software platform (Cauz-Santos et al., 2017). The measure of RSCU, defined as the ratio of the frequency of a particular codon to the expected frequency of that codon, was utilized (Sharp and Li, 1987). A value exceeding 1 indicates that a codon is utilized more frequently than anticipated, while a value below 1 signifies less frequent usage than expected. This approach provides insights into the codon usage patterns within the chloroplast genomes under investigation.

### 2.5 Simple sequence repeat (SSR) analysis

Chloroplast SSRs in the chloroplast genome sequences of *Lagerstroemia* “Pink Velour” were identified using the MISA software (Liu et al., 2018). The criteria for minimum numbers of repeats were set as follows: the repeats included mono-, di-, tri-, tetra-, penta-, and hexanucleotides with a minimum of 10, 5, 4, 3, 3, and three repeats, respectively.

### 2.6 Evolutionary and phylogenetic analyses

Phylogenetic relationships among *Lagerstroemia* species closely related to *L. indica* were analyzed based on the complete chloroplast genomes of 26 *Lythraceae* species, including 23 *Lagerstroemia* species. Sequences were aligned using MAFFT (Katoh, 2005). For maximum likelihood analysis, the GTRGAMMA model was used for all datasets, and self-expanding analyses were conducted with 1,000 repetitions (Stamatakis et al., 2008). This study aims to elucidate the evolutionary mechanisms underlying the closely related species of *L. indica*. The seven tested varieties of *Lagerstroemia* species exhibit close genetic affinities yet display distinct phenotypic traits (www.iplant.cn) (Pooler, 2003; Choi et al., 2010). The seven varieties possess rich genetic diversity, making them ideal candidates for further molecular analysis to investigate the evolutionary mechanisms among *Lagerstroemia* relatives.

### 2.7 Chloroplast genome comparison and IR border and divergence analyses

The chloroplast genomes of seven *Lagerstroemia* species were aligned using mVISTA with the LAGAN alignment program (Brudno et al., 2003). *Lagerstroemia* “Pink Velour” served as the reference sequence. The chloroplast genomes of the six remaining species were obtained from the NCBI based on homologous alignment (*L. indica* PP404026, *Lagerstroemia caudata* NC_060355, *Lagerstroemia excelsa* NC_042896, *Lagerstroemia fauriei* MT019854, *Lagerstroemia limii* NC_042889, *Lagerstroemia subcostata* NC_034952, *Lagerstroemia suprareticulata* NC_071782). The boundaries of LSC-IRb, IRb-SSC, and SSC-IRa in the chloroplast genomes were compared and visualized using the online platform CPJSdraw (http://cloud.genepioneer.com:9929/#/tool/alltool/detail/296) (Shi et al., 2023). To identify mutation hotspot regions and genes, the complete chloroplast genome sequences of the seven *Lagerstroemia* species were aligned using the MAFFT tool (Katoh and Standley, 2013). Nucleotide diversity (Pi) values for highly variable regions were calculated by DnaSP5 (http://www.ub.edu/dnasp/) (Kang et al., 2019). DNAMAN software was utilized for nucleic acid sequence alignment. DnaSP 5.1 (Librado and Rozas, 2009; Guo et al., 2016) was employed to calculate nonsynonymous substitutions (Ka), synonymous substitutions (Ks), and their ratio (Ka/Ks). The Ka/Ks value cannot be calculated if Ks = 0.

## 3 Results

### 3.1 Assembly of the chloroplast genome with a length of 162,174 base pairs in *Lagerstroemia* “Pink Velour”

**FIGURE 1:**
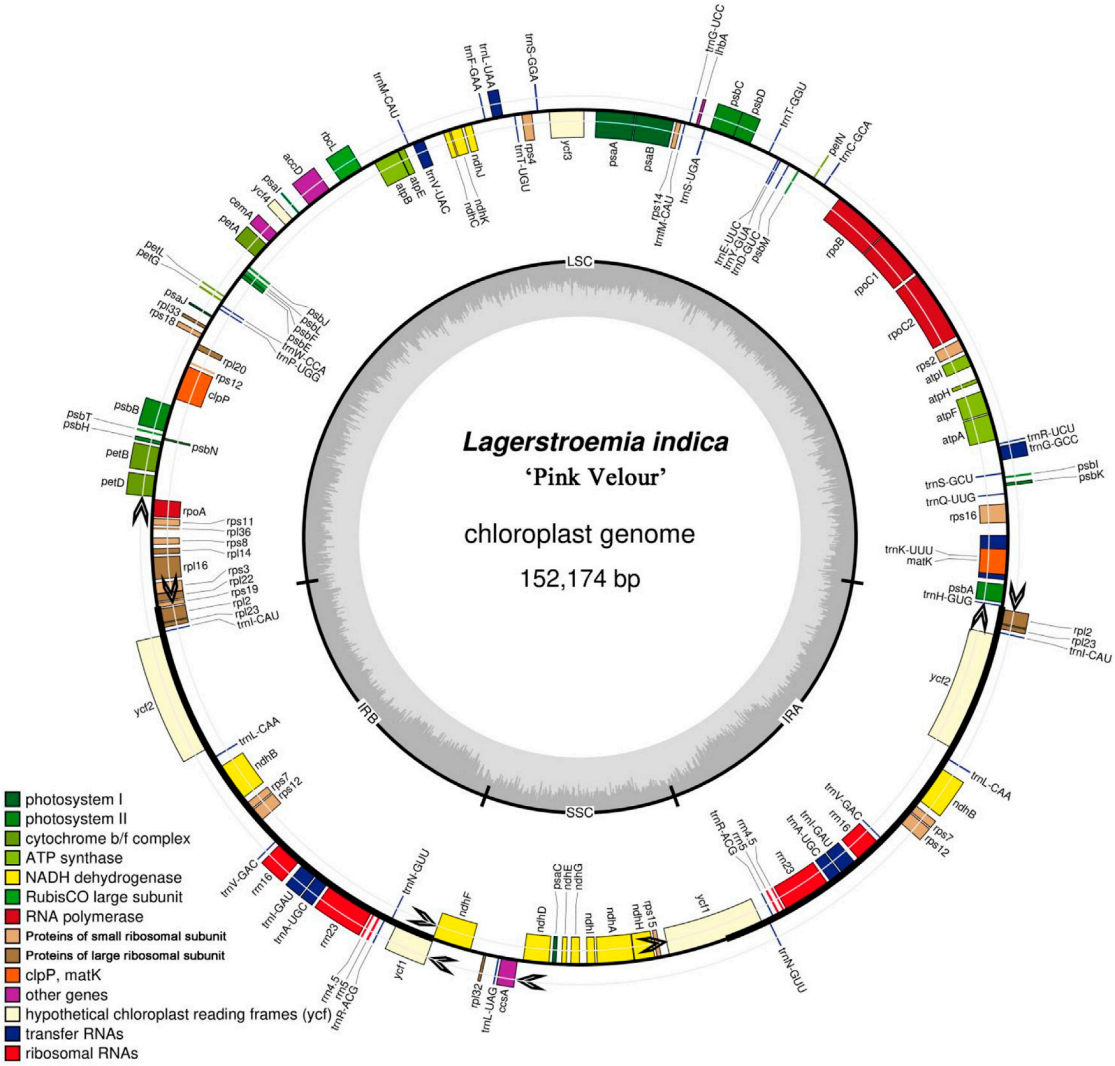
Gene map of the *Lagerstroemia* “Pink Velour” chloroplast genome. The gene map provides a comprehensive overview of the complete chloroplast genome of *Lagerstroemia* “Pink Velour”. In chloroplast genome, the black arrows represent the transcriptional direction. Genes outside the black circle are transcribed clockwise, while those inside are transcribed counterclockwise. The inner black circle denotes the boundaries of the LSC, SSC, IRa and IRb regions. The inner circle’s dark grey shading indicates the GC content, while the light grey represents the AT content. On the outer circle, distinct blocks of various colors symbolize different functional groups of genes.

The *Lagerstroemia* “Pink Velour” chloroplast genome, spanning 152,174 bp, displays a classical cyclic double-stranded four-region structure. This structure encompasses a pair of inverted repeats (IRs, 51,250 bp), a large single-copy region (LSC, 84,006 bp), and a small single-copy region (SSC, 16,918 bp). The GC content across these regions was observed to be 39.50% (Supplementary Table S1). Comprising a total of 130 chloroplast RNAs, the genome includes 85 protein-coding genes, 37 tRNA genes, and 8 rRNA genes. These annotated genes fall into four categories, namely, genes associated with photosynthesis, self-replication, other known functions, and genes with unknown functions (Figure 1; Table 1). Notably, 18 of these genes were present in duplicate within the IR regions, encompassing 5 protein-coding genes (*rps7*, *rps12*, *ndhB*, *rpl2*, and *rpl23*), seven tRNA genes (*trnN-GUU*, *trnA-UGC*, *trnV-GAC*, *trnL-CAA*, *trnI-CAU*, *trnI-GAU*, and *trnR-ACG*), 4 rRNA genes (*rrn4.5*, *rrn5*, *rrn16*, and *rrn23*), and two conserved hypothetical chloroplast ORF genes (*ycf1* and *ycf2*). In total, 17 genes (*petB*, *petD*, *ndhA*, *ndhB*, *atpF*, *rpl16*, *rps12*, *rps16*, *rpoC1*, *trnA-UGC*, *trnG-GCC*, *trnI-GAU*, *trnK-UUU*, *trnL-UAA*, *trnV-UAC*, *clpP*, and *ycf3*) with 1 or two introns were identified (Table 1). Among these intron-containing genes, 13 (*petB*, *petD*, *atpF*, *rpl16*, *trnG-GCC*, *trnK-UUU*, *trnL-UAA*, *trnV*, *UAC*, *rps16*, *rpoC1*, *rps12*, *clpP*, and *ycf3*) were located in the LSC region, one (*ndhA*) in the SSC region, and three (*ndhB*, *trnA-UGC*, and *trnI-GAU*) in both IR regions. Noteworthy is the positioning of the *matK* gene within the intron of *trnK-UUU* (Figure 1). The full names of genes in the artical were listed in Supplementary Table S2.

**TABLE 1:** List of annotated genes in the chloroplast genome of *Lagerstroemia* “Pink Velour”.

Category	Gene group	Gene name
Photosynt-hesis Related Genes	Subunits of Photosystem I	*psaA*, *psaB*, *psaC*, *psaI*, *psaJ*
	Subunits of Photosystem Ⅱ	*psbA*, *psbB*, *psbC*, *psbD*, *psbE*, *psbF*, *psbH*, *psbI*, *psbJ*, *psbK*, *psbL*, *psbM*, *psbN*, *psbT*
	Subunits of NADH Dehydrogenase	ndhA*, ndhB*(2), *ndhC*, *ndhD*, *ndhE*, *ndhF*, *ndhG*, *ndhH*, *ndhI*, *ndhJ*, *ndhK*
	Subunits of Cytochrome b/f Complex	*petA*, petB*, petD*, *petG*, *petL*, *petN*
	Subunits of ATP Synthase	*atpA*, *atpB*, *atpE*, atpF*, *atpH*, *atpI*
	Large Subunit of Rubisco	*rbcL*
Self-replication	Proteins of Large Ribosomal Subunit	rpl14, rpl16*, *rpl2(2)*, *rpl20*, *rpl22*, *rpl23(2)*, *rpl32*, *rpl33*, *rpl36*
	Proteins of Small Ribosomal Subunit	*rps11*, rps12**(2), *rps14*, *rps15*, rps16*, *rps18*, *rps19*, *rps2*, *rps3*, *rps4*, *rps7(2)*, *rps8*
	Subunits of RNA Polymerase	*rpoA*, *rpoB*, rpoC1*, *rpoC2*
	Ribosomal RNAs	*rrn16(2)*, *rrn23(2)*, *rrn4.5(2)*, *rrn5(2)*
	Transfer RNAs	trnA-UGC*(2), *trnC-GCA*, *trnD-GUC*, *trnE-UUC*, *trnF-GAA*, trnG-GCC*, *trnG-UCC*, *trnH-GUG*, *trnI-CAU(2)*, trnI-GAU*(2), trnK-UUU*, *trnL-CAA(2)*, trnL-UAA*, *trnL-UAG*, *trnM-CAU*, *trnN-GUU(2)*, *trnP-UGG*, *trnQ-UUG*, *trnR-ACG(2)*, *trnR-UCU*, *trnS-GCU*, *trnS-GGA*, *trnS-UGA*, *trnT-GGU*, *trnT-UGU*, *trnV-GAC(2)*, trnV-UAC*, *trnW-CCA*, *trnY-GUA*, *trnfM-CAU*
Other Genes	Maturase	*matK*
	Protease	clpP**
	Envelope Membrane Protein	*cemA*
	Acetyl-CoA Carboxylase	*accD*
	c-type Cytochrome Synthesis Gene	*ccsA*
Genes of Unknown Function	Conserved Hypothetical Chloroplast ORF	*lhbA*, *ycf1(2)*, *ycf2(2)*, ycf3**, *ycf4*

^a^ Gene*: contains an intron; Gene**: contains two introns. Gene (2): has two copies.

### 3.2 The most abundant SSR type: T or A base repeats

**FIGURE 2:**
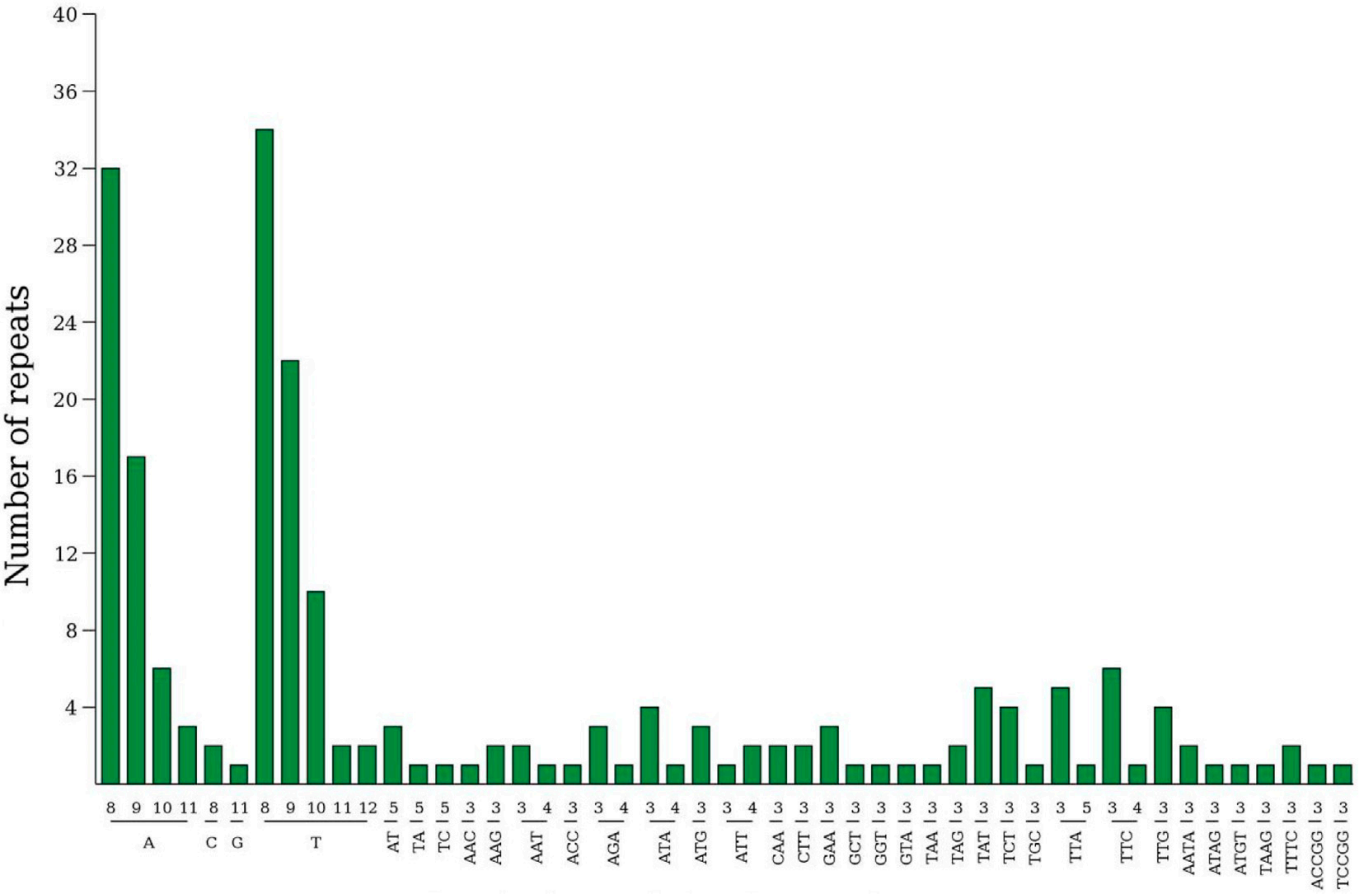
Analysis of simple sequence repeats (SSRs) in *Lagerstroemia* “Pink Velour”. The horizontal axis illustrates the length of the repeats and the corresponding repeated sequences, while the vertical axis quantifies the number of repeats for each identified category. The numbers on the *x*-axis represent the number of base repeats.

A total of 207 simple sequence repeats (SSRs) were identified within the *Lagerstroemia* “Pink Velour” chloroplast genome (Supplementary Table S3; Figure 2). The predominant SSR type was single nucleotide repeats, constituting the majority at 63.28%. Trinucleotide repeats (29.95%), 4-nucleotide repeats (3.38%) and 2-nucleotide repeats (2.41%) were the next most abundant SSR types, while 5-nucleotide repeats were the least abundant (0.96%). Within the mononucleotide repeats, A/T repeats emerged as the most prevalent type, with repeat numbers ranging from 8 to 12 in this investigation. The most abundant SSR type in seven *Lagerstroemia* species is TTTTTTTT (T8). The second most abundant SSR type is AAAAAAAA (A8).

### 3.3 Preference for AT base termination in codon pairs

**FIGURE 3:**
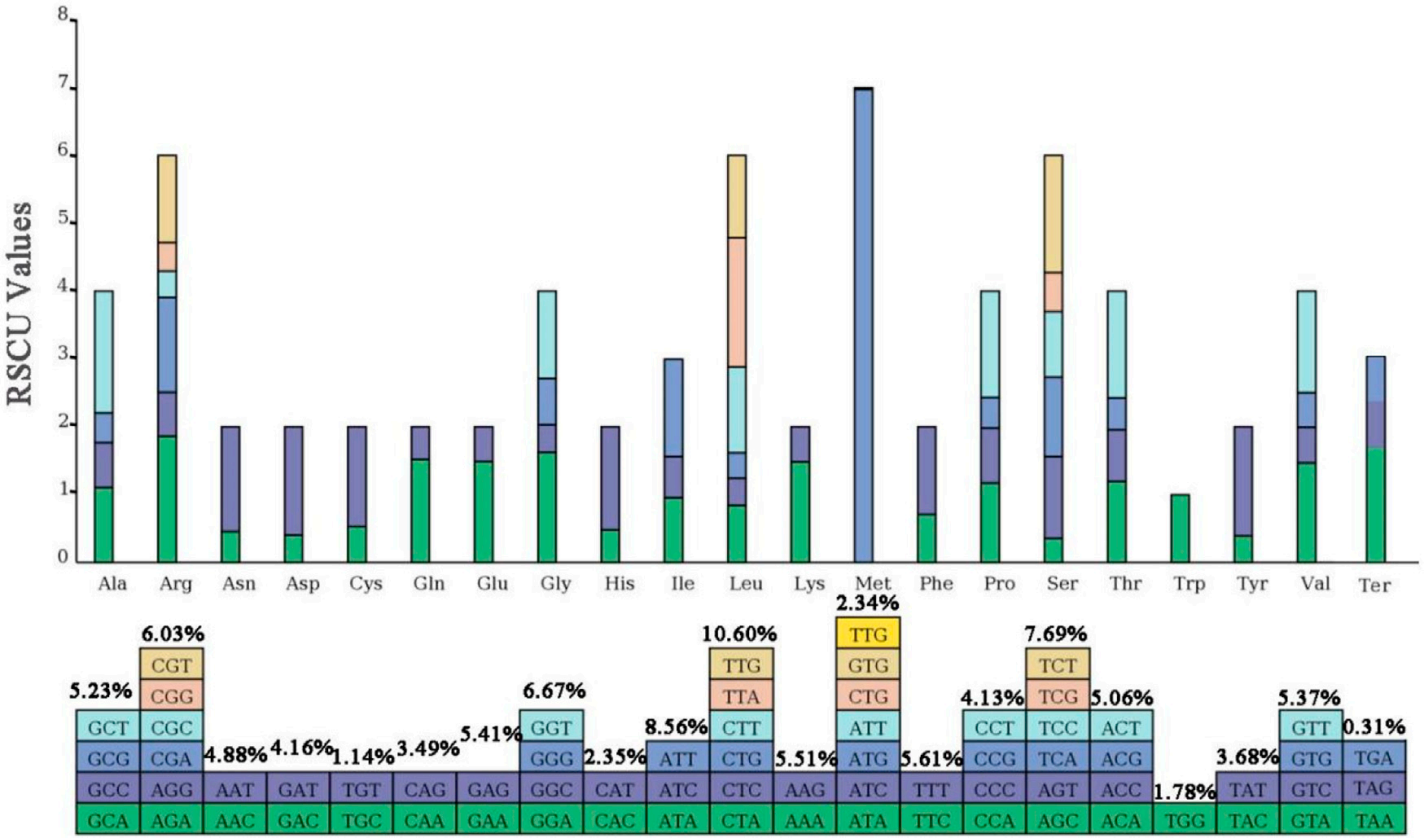
RSCU analysis of *Lagerstroemia* “Pink Velour”. Each square corresponds to all codons encoding a specific amino acid. The height of the upper column indicates the sum of RSCU values for all codons associated with that amino acid. The number above the codons represent the frequency of use of all codons corresponding to this amino acid.

The relative synonymous codon usage (RSCU) of the chloroplast genome of *Lagerstroemia* “Pink Velour” was conducted using all protein-coding genes, encompassing a total of 27,041 codons (Figure 3; Supplementary Table S4). Leucine (Leu), encoded by CTA, CTC, CTG, CTT, TTA, and TTG, emerged as the most abundant amino acid, constituting 10.60% (2,865 codons) of the total codons. Isoleucine (Ile), encoded by ATA, ATC, and ATT, ranked as the second most abundant amino acid, representing 8.56% (2,316 codons). Conversely, Termination (Ter), encoded by TAA, TGA, and TAG, was the least frequently encoded amino acid, accounting for a mere 0.31% (85 codons) of the total. RSCU, mitigating the impact of amino acid composition on codon usage effectively, revealed that a majority of codons with RSCU values greater than 1 concluded with A or T. A total of 31 codons exhibited RSCU values exceeding 1, with the highest value observed for ATG at 6.97. And Methionine (Met) encoded by the codons ATG (631), ATT (1) and GTG (1), and comprises 2.34% of the total codon population. The attachment Supplementary Table S4 shows the counts and frequencies of usage for all codons.

### 3.4 Phylogenetic analysis involving 26 Lythraceae related species

**FIGURE 4:**
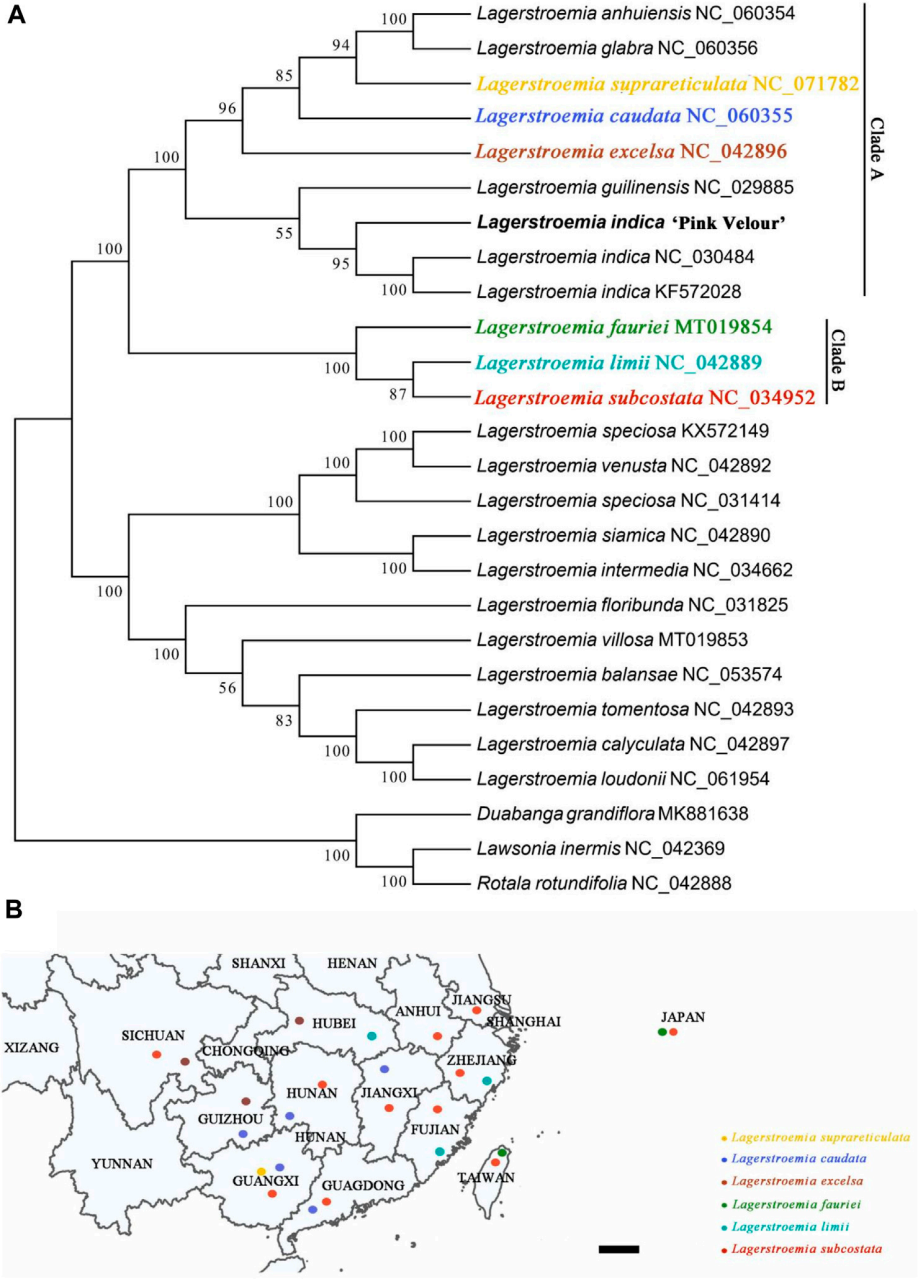
A phylogenetic tree of 26 *Lagerstroemia* species. **(A)** The phylogenetic tree was constructed by the ML method and complete chloroplast genome sequences. Bootstrap values from 1,000 replicates are shown near the branches. GenBank accessions are provided after the species name. The *Lagerstroemia* “Pink Velour” sequence obtained in this study is highlighted in bold *Lagerstroemia indica*. **(B)** Geographic distributions of the six *Lagerstroemia* species. Yellow circle represents species *Lagerstroemia suprareticulata*. Blue circle represents species *Lagerstroemia caudata*. Brown circle represents species *Lagerstroemia excelsa*. Green circle represents species *Lagerstroemia fauriei*. Turquoise circle represents species *Lagerstroemia limii*. Red circle represents species *Lagerstroemia subcostata.* Scale = 20 KM.

Utilizing the chloroplast genome, we conducted a phylogenetic analysis involving 26 related species. The objective was to elucidate the phylogenetic relationships existing among the examined species and their proximate relatives. Additionally, we sought to ascertain their systematic positions within the Lythraceae family (Figure 4A). The results showed that the newly obtained sequence of *Lagerstroemia* “Pink Velour” clustered with *L. indica* (NC-030484) and *L. indica* (KF572028) and differentiated earlier than these two *L. indica* samples. In addition, the phylogenetic tree constructed based on the chloroplast genome confirmed that *L. indica* is closely related to *Lagerstroemia guilinensis* but is more distantly related to *L. excelsa*, *L. caudata*, *L. suprareticulata*, *Lagerstroemia glabra*, and *Lagerstroemia anhuiensis*. The native habitats of seven *Lagerstroemia* species are shown in Figure 4B.

### 3.5 Variation in the boundary of the JSA harboring the *ycf1* gene among seven *Lagerstroemia* species chloroplast genomes

**FIGURE 5:**
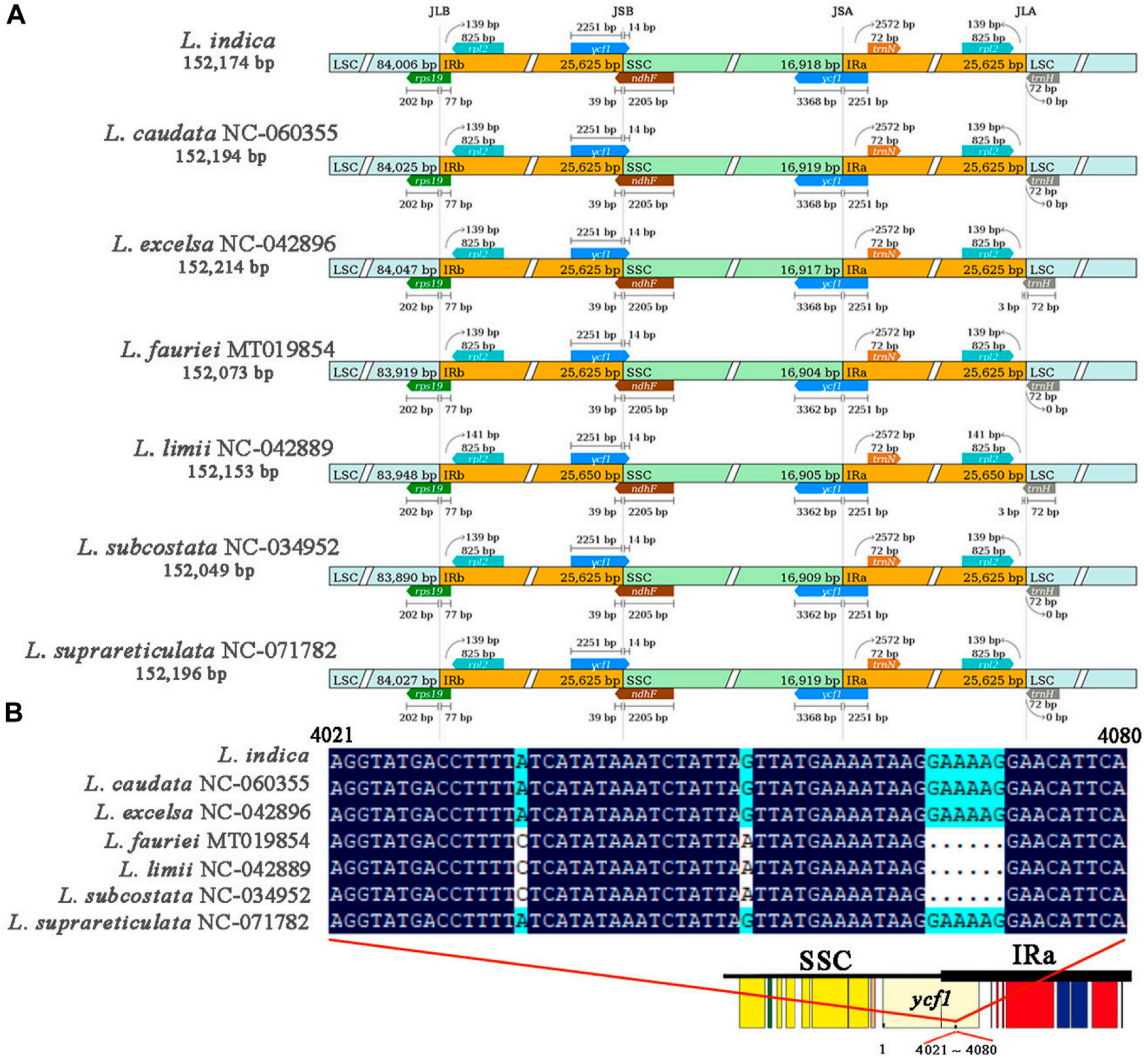
Comparison of the positions of the LSC, SSC, and IR region borders among 7 *Lagerstroemia* species. **(A)** Genes are represented by boxes, and the distance between genes and their boundaries is indicated by the number of bases, unless the gene extends to the boundary. The extensions of genes are also indicated above the boxes. **(B)** Comparison of the gene *ycf1* crossing the JSA boundaries in 7 *Lagerstroemia* species. Schematic diagram showing the location of the *ycf1* gene on the chloroplast genome, with a deletion of six bases in the 4,021 to 4,080 base range of the *ycf1* gene in species *Lagerstroemia fauriei, Lagerstroemia limii* and *Lagerstroemia subcostata.*

The results of collinearity analysis of the chloroplast genomes of 7 *Lagerstroemia* species (*L. indica* PP404026, *L. caudata* NC_060355, *L. excelsa* NC_042896, *L. fauriei* MT019854, *L. limii* NC_042889, *L. subcostata* NC_034952, *L. suprareticulata* NC_071782) showed that the gene order of all chloroplast genomes was consistent, and no rearrangement or inversion occurred (Supplementary Figure S1). The boundary positions of the chloroplast genomes of the 7 *Lagerstroemia* species were comprehensively compared (Figure 5A). The results showed that these chloroplast genomes were highly conserved, while there were also some differences. The LSC/IRb (JLB), IRb/SSC (JSB) and SSC/IRa (JSA) boundaries were located in the *rps19*, *ycf1* and *ycf1* genes, respectively. The IRa/LSC (JLA) boundary was located between the *rpl2* and *trnH* genes. The *rps19* gene spans the JLB boundary to the IRb region at 77 bp, the *ycf1* gene spans the JSB boundary to the IRb region at 2,251 bp, the *ycf1* gene spans the JSA boundary to the IRa region at 2,251 bp, the *rpl2* gene is 139–141 bp away from the JLA boundary, and *trnH* spans the JLA boundary to the IRa region at 0–3 bp. It is worth noting that the length of the cross-domain gene *ycf1* varies, which is caused by *L. fauriei*, *L. limii*, and *L. subcostata* lacking six bases between the 4,021 and 4,080 sites in the full length of *ycf1* (Figure 5B).

### 3.6 High mutation sites observed in the LSC region of seven *Lagerstroemia* species chloroplast genomes

**FIGURE 6:**
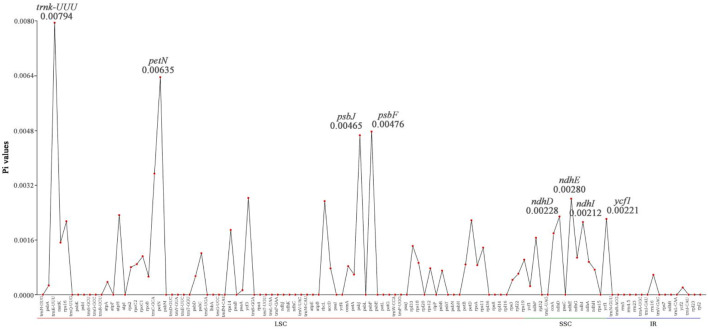
Comparison of nucleotide diversity (Pi) values among 7 *Lagerstroemia* species. The abscissa represents 113 genes distributed across the LSC, SSC, and IR regions of the chloroplast genome, while the ordinate represents the gene Pi values.

In this study, the analysis of nucleotide diversity showed that the average nucleotide diversity of the seven species was only 0.00068. However, the nucleotide diversity showed significant differences among regions of the chloroplast genome, with the nucleotide diversity in the LSC region ranging from 0 to 0.0079, with an average of 0.00072. The nucleotide diversity of the SSC region ranged from 0 to 0.0028, with an average of 0.0011. The nucleotide diversity of the IR regions ranged from 0 to 0.00058, with an average of only 0.000049. The nucleotide diversity of the SSC region in the chloroplast genomes of the three species was the highest, while the nucleotide diversity of the IR regions was lower than that of the other two regions (Figure 6), indicating that the IR regions are more conserved than the other two regions. The above results suggested that there is little variation among the chloroplast genomes of the seven species. However, there are still highly variable sites (Pi > 0.004), such as *trnK-UUU*, *petN*, *psbJ*, and *psbF,* in the chloroplast genomes of the seven species. These sites are all distributed in the LSC region of the chloroplast genome.

**FIGURE 7:**
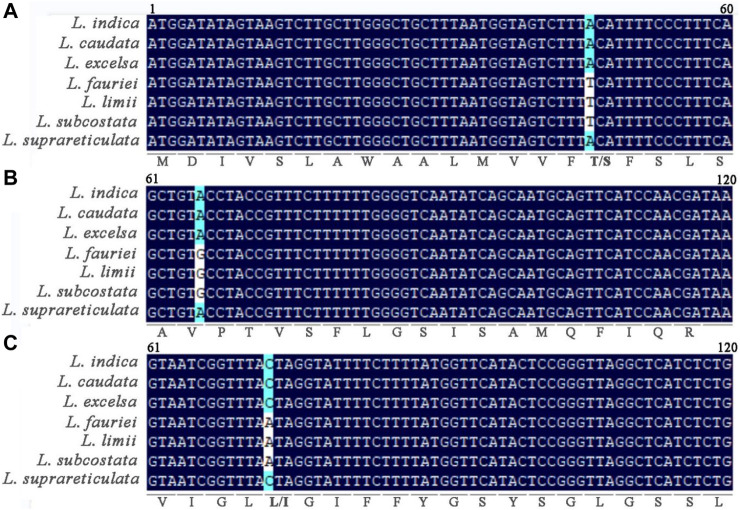
Nucleotide mutations in *petN* **(A)**, *psbJ* **(B)**, and *psbF* **(C)** in 7 *Lagerstroemia* species. The numbers represent the position of the gene fragment on the corresponding gene. The letters below the bases represent the translated amino acids. A (Ala, ALanine); R (Arg,Arginine); D (Asp, Aspartic acid); Q (Gln, Glutamine); G (Gly, Glycine); I (Ile, Isoleucine); L (Leu, Leucine); M (Met, Methionine); F (Phe, Phenylalanine); P (Pro, Proline); S (Ser, Serine); T (Thr, Threonine); W (Trp, Tryptophan); Y (Tyr, Tyrosine); V (Val, Valine).

Single-nucleotide variants in chloroplast genome genes were compared within *Lagerstroemia* (Figure 7). In the *petN* gene, a thymine (T) was shared among *L. fauriei*, *L. limii*, and *L. subcostata*, while at this same position in the other three *Lagerstroemia* species sampled, adenine (A) was observed. In the *psbJ* gene, guanine (G) and A were found in different *Lagerstroemia* species. In the *psbF* gene, variation between A and cytosine (C) was also found among these species. These differences result in synonymous or nonsynonymous changes in amino acids. For example, a codon of serine (S) in the *L. fauriei*, *L. limii*, and *L. subcostata* chloroplast genomes was changed to threonine (T). Unlike in the *petN* and *psbF* genes, no amino acid translation by variable codons was observed in *psbJ*. This suggests that these changes may be related to the evolution of chloroplast gene functions in *Lagerstroemia* species.

### 3.7 Higher nonsynonymous substitutions occurrence of photosynthesis genes among *Lagerstroemia fauriei*, *Lagerstroemia limii* and *Lagerstroemia subcostata*

The Ka/Ks ratio, which compares the frequency of nonsynonymous nucleotide substitutions with synonymous substitutions, serves as a crucial metric in assessing selective constraints on gene diversification. Values exceeding 1 suggest that genes are influenced by positive selection, a ratio of 1 indicates neutral selection, while ratios less than 1 imply purifying selection. We calculated the Ka and Ks values for genes in the chloroplast genomes of 6 *Lagerstroemia* species with that of *L. indica* “Pink Velour” as the reference. The Ka/Ks ratios in *Lagerstroemia* species ranged from 0.00000 to 1.59332. Among these genes, only 14 had calculable Ka/Ks values (Supplementary Table S5). Among the genes with base substitution, the Ks values of 12 genes were greater than the Ka values. The Ks values of two genes (*petD* and *rbcL*) were lower than the Ka values, yielding Ka/Ks values > 1.

**FIGURE 8:**
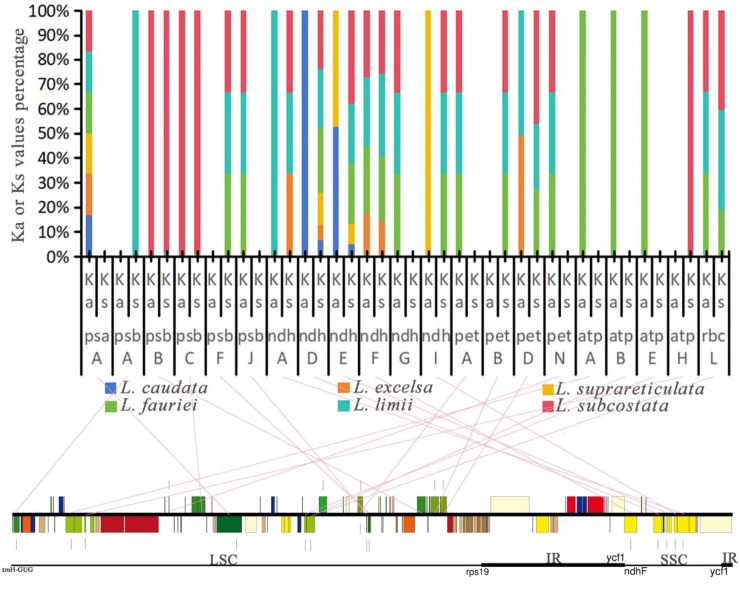
Ka or Ks value percentage for photosynthesis genes in the chloroplast genomes of 6 *Lagerstroemia* species in comparison with that of *Lagerstroemia indica* “Pink Velour”. Pink straight lines connect the photosynthesis genes with their locations on the chloroplast genome.

We calculated the Ka or Ks value percentage for 20 photosynthesis genes in six species, and values equal to 0 indicated that these gene sequences were conserved without synonymous nucleotide substitutions (Figure 8). The results revealed that 17 genes underwent nonsynonymous nucleotide substitution, while 13 genes underwent synonymous nucleotide substitution. Nine genes underwent synonymous or nonsynonymous substitution in only 1 species. The genes exhibiting synonymous or nonsynonymous substitutions exclusively in three species include *psbF*, *psbJ*, *ndhG*, *ndhI*, *petA*, *petB*, *petN*, and *rbcL*. These genes fall under the categories of “subunits of photosystem II”, “subunits of NADH dehydrogenase”, “subunits of cytochrome b/f complex” and “large subunit of rubisco”. On the other hand, *psaA* and *ndhD*, categorized as “subunits of photosystem I″ and “subunits of NADH dehydrogenase”, respectively, underwent synonymous or nonsynonymous substitutions in all six species.

## 4 Discussion

In the present study, we conducted the sequencing of the chloroplast genome of *L. indica*, yielding a circular genome of 152,174 base pairs with a quadripartite structure similar to that in other *Lagerstroemia* species, such as *Lagerstroemia intermedia* (Gu et al., 2017). Analyses of chloroplast genome structure, SSRs, codon usage, sequence divergence, nucleotide polymorphism, and evolutionary selection were performed on related *Lagerstroemia* species. The results prove that slight differences exist among these six species. Differences between these closely related species were observed, primarily involving the *ycf1* gene at the boundary in JSA. Further investigation of individual nucleotide substitution and Ka (Ks) value proportions revealed evolutionary selection among closely related species of *Lagerstroemia*.

The chloroplast genome of *Lagerstroemia* “Pink Velour” was consistent with those of other species, such as *L. caudata*, *L. excelsa*, *L. fauriei*, and *L. limii,* in terms of gene structure (Xu et al., 2017; Gu et al., 2019; Zheng et al., 2020; Dong et al., 2021). The AT content in the *Lagerstroemia* “Pink Velour” chloroplast genome was found to be higher than the GC content, similar to the previous studies (Gu et al., 2019; Zheng et al., 2020). Notably, the GC content exhibited variations among the IR and single-copy regions, potentially linked to the presence of rRNA genes within the IR regions (Zheng et al., 2020). In our study, codons with A or T at the 3′position demonstrated higher RSCU values (≥1) (Figure 3). This outcome could be attributed to the elevated AT content commonly observed in plant chloroplast genomes (Menezes et al., 2018). Similar patterns in codon usage have been reported in other plant species (Li et al., 2019; Peng et al., 2022). Furthermore, The most common repeat units in all SSRs were A/T (Figure 2). This observation parallels findings from earlier studies (Ping et al., 2021; Fan et al., 2022). The abundance of A/T repeat units may be linked to their concentration in non-coding regions characterized by lower GC contents. SSRs hold potential utility in plant classification studies and can serve as molecular markers in phylogenetic investigations (Abdullah et al., 2021; Yang et al., 2021).

The boundaries between the LSC IRa, SSC, and IRb regions hold significance in the evolution of certain taxa (Nazareno et al., 2015). The presence of the ycf1 pseudogene was identified in all seven species, yet some nucleic acid sequences were absent in three species (Figure 5). The observed variation was concentrated within the IR regions. Nucleic acid sequence variations in ycf1 were detected in the 7 *Lagerstroemia* chloroplast genome sequences. The sequence expansion in the *Lagerstroemia* transcribed region was regarded as a significant evolutionary event in *L. indica*, *L. caudata*, *L. excelsa*, and *L. suprareticulata*. *Ycf1* variation among these seven species may have arisen 20.1 million years ago (Wang et al., 2023).

Nucleotide diversity (Pi) serves as an indicator of variation in nucleic acid sequences, providing a valuable molecular marker for population genetics (Mehmood et al., 2020; Ding et al., 2021). Established methods for constructing DNA barcodes, as demonstrated in other species (Ali et al., 2020), have been applied in this study. The Pi analysis unveiled that the IR region exhibited higher conservation compared to the LSC and SSC regions (Figure 6). Furthermore, protein-coding genes such as *trnk-UUU*, *pet-N*, *psbJ*, *psbF*, *ndhE*, *ndhD*, *ndhI*, *and ycf1* were found to have larger variations in the 7 *Lagerstroemia* plastomes, and these genes with variation sites were divided into two clades (Figure 4). Most of the genes with higher Pi values in this study belonged to the photosynthesis category, which was different from previous findings in *Lagerstroemia* species (Gu et al., 2019; Ping et al., 2021). Life history differences and biological, and nonbiological stressors lead to variation in plastome evolution (Bousquet et al., 1992; Ge and Guo, 2020), and the kinship of species was also an influencing factor in this study. In subsequent investigations, pet-N, psbJ, and psbF stand out as potential molecular markers that can be explored for their efficacy in elucidating the phylogenetic relationships among various closely related species (Figure 7). Synonymous or non-synonymous mutations caused by variation in a single nucleotide may result from natural selection.

The first and second codon positions are often more informative than the third position for gene function changes, as they are usually synonymous (Wang et al., 2023). The synonymity of most photosynthesis genes in this study suggests adaptation to novel ecological conditions, such as temperature, light, and moisture (Zheng et al., 2020). Most photosynthesis genes are synonymous or non-synonymous only in the species *L. fauriei*, *L. limii*, and *L. subcostata* (Figure 8). These genes found in six species suggests a potentially crucial role in their adaptation to diverse selective environments. The origin of these species is divided into the islands and the continent (Figure 4B), the biological and nonbiological stressors they received may provid environmental conditions for photosynthesis genes evolution. In addition, previous research results have shown that deletion of *petG* or *petN* caused a bleached phenotype and loss of photosynthetic electron transport and photoautotrophy (Schwenkert et al., 2007). When *psbJ* is absent, both intact PSII core monomers and PSII core dimers containing the PsbO protein are formed. However, the LHCII antenna remains dissociated from the PSII dimers (Suorsa et al., 2004). The nuclear-encoded *PBR1* exerts tight control over the translational expression of the chloroplast gene *Ycf1*, thereby facilitating the coordinated biogenesis of the NDH, PSI, and Cytb6f complexes as a unit (Yang et al., 2016). The *orrm6* mutants exhibit reduced levels of photosystem II (PSII) proteins, particularly *PsbF*, along with diminished PSII activity, pale green pigmentation, smaller leaf and plant sizes, and delayed growth (Hackett et al., 2017). The measurements of photosynthetic physiological indices (including chlorophyll fluorescence indices, chlorophyll content, vegetative growth of plants, etc.) were included in these studies, which were lacking in our research. Future experiments need further exploration and investigation to determine the functional changes of mutated genes.

Chloroplast genome sequence data have been extensively used in plant phylogenetic reconstruction (Liu et al., 2018). In the present study, we performed phylogenetic analysis based on *Lagerstroemia* chloroplast genomes in relation to those reported for species (Figure 4). The results strongly supported *L. indica* and *L. guilinensis* as homologous, consistent with the findings in prior studies (Zheng et al., 2020; Ping et al., 2021). We also found that the species in clades A and B were closely related, and the relationships of *L. suprareticulata* and *L. caudata* were reported in detail for the first time. The results could provide a background reference for *Lagerstroemia* breeding and even accelerate horticultural breeding through molecular marker-assisted selection.

## 5 Conclusion

In this study, the chloroplast genome of *L. indica* “Pink Velour” was sequenced and assembled. Following this, a comparative chloroplast genome analysis unveiled that the structural features of the *Lagerstroemia* “Pink Velour” genome showed differences in JSA boundary. These findings offer novel insights into unraveling the phylogenetic relationships among related *Lagerstroemia* species. Identification of single-nucleotide variants within *Lagerstroemia* species revealed that photosynthesis genes play an important role in molecular evolution. Highly variable genes have been identified that can be used for species differentiation and marker-assisted breeding. The variation of photosynthesis genes provides a deeper understanding of the evolutionary mechanism, which provides information on climatic adaptation in important ornamental plants.

## Data Availability

The data presented in the study are deposited in the NCBI (National Center for Biotechnology Information) repository, accession number PP404026; for more information regarding our data policies, refer to our guidelines.
