# Supplementary material for: Chloroplast genomes in seven Lagerstroemia species provide new insights into molecular evolution of photosynthesis genes
Source: Front Genet. 2024 Apr 2;15:1378403. doi: 10.3389/fgene.2024.1378403 (PMC11019025; doi:10.3389/fgene.2024.1378403)
Supplement: Supplementary file 1 [file Table1.DOCX]

| Unit size(1) | Unit size(2) | Unit size(3) | Unit size(4) | Unit size(5) | Total SSRs | LSC | SSC | IRs |
| --- | --- | --- | --- | --- | --- | --- | --- | --- |
| 131 | 5 | 62 | 7 | 2 | 207 | 128 | 35 | 44 |

Supplementary Material

| Genome size (bp) | LSC length (bp) | SSC length (bp) | IR length (bp) | LSC GC content (%) | SSC GC content (%) | IR GC content (%) | Total GC content (%) |
| --- | --- | --- | --- | --- | --- | --- | --- |
| 152,174 | 84,006 | 16,918 | 51,250 | 35.94 | 31.00 | 42.50 | 37.60 |

**Table S1.** Summary of the chloroplast genome features of *Lagerstroemia indica* cv. Ebony Embers

**Table S2.** Number of simple sequence repeats (SSRs) in chloroplast genomes of *Lagerstroemia indica* cv. Ebony Embers


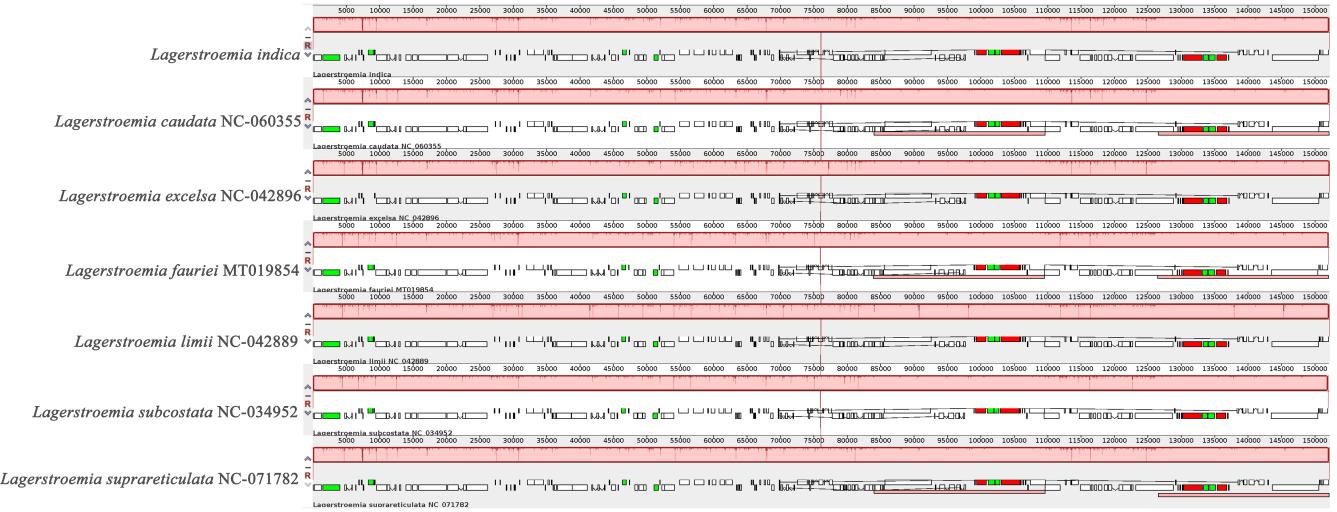


**Figure S1**. Genome rearrangement of 7 Lagerstroemia species.
